# Phase II multicenter randomized controlled clinical trial on the efficacy of intra-articular injection of autologous bone marrow mesenchymal stem cells with platelet rich plasma for the treatment of knee osteoarthritis

**DOI:** 10.1186/s12967-020-02530-6

**Published:** 2020-09-18

**Authors:** José María Lamo-Espinosa, Juan F. Blanco, Mikel Sánchez, Victoria Moreno, Froilán Granero-Moltó, Fermín Sánchez-Guijo, Íñigo Crespo-Cullel, Gonzalo Mora, Diego Delgado San Vicente, Orlando Pompei-Fernández, Jesús Dámaso Aquerreta, Jorge María Núñez-Córdoba, María Vitoria Sola, Andrés Valentí-Azcárate, Enrique J. Andreu, María del Consuelo del Cañizo, Juan Ramón Valentí-Nin, Felipe Prósper

**Affiliations:** 1 Department of Orthopaedic Surgery and Traumatology, Clínica Universidad de Navarra, 36 Pío XII Avenue, 31008 Pamplona, Spain; 2 Cell Therapy Area, Clínica Universidad de Navarra, 36 Pío XII Avenue, 31008 Pamplona, Spain; 3 Department of Orthopaedic Surgery and Traumatology, Complejo Universitario de Salamanca-IBSAL, Salamanca, Spain; 4 Arthroscopic Surgery Unit, Hospital Vithas San José, Vitoria‐Gasteiz, Spain; 5 Advanced Biological Therapy Unit, Hospital Vithas San José, Vitoria‐Gasteiz, Spain; 6 Department of Haematology, Complejo Hospitalario de Salamanca-IBSAL, Salamanca, Spain; 7 Department of Radiology, Clínica Universidad de Navarra, Pamplona, Spain; 8 Division of Biostatistics, Research Support Service, Central Clinical Trials Unit, Clínica Universidad de Navarra, Pamplona, Spain; 9 Department of Preventive Medicine and Public Health, Medical School, University of Navarra, Pamplona, Spain; 10 Department of Haematology, Clínica Universidad de Navarra, Pamplona, Spain

**Keywords:** Bone marrow-mesenchymal stromal cells, Knee osteoarthritis, Non-surgical management, Stem cell therapy

## Abstract

**Background:**

Mesenchymal stromal cells are a safe and promising option to treat knee osteoarthritis as previously demonstrated in different clinical trials. However, their efficacy, optimal dose and addition of adjuvants must be determined. Here, we evaluated the clinical effects of a dose of 100 × 10^6^ bone marrow mesenchymal stromal cells (BM-MSCs) in combination with Platelet Rich Plasma (PRGF®) as adjuvant in a randomized clinical trial.

**Methods:**

A phase II, multicenter, randomized clinical trial with active control was conducted. Sixty patients diagnosed with knee OA were randomly assigned to 3 weekly doses of PRGF® or intraarticular administration of 100 × 10^6^ cultured autologous BM-MSCs plus PRGF®. Patients were followed up for 12 months, and pain and function were assessed using VAS and WOMAC and by measuring the knee range of motion range. X-ray and magnetic resonance imaging analyses were performed to analyze joint damage.

**Results:**

No adverse effects were reported after BM-MSC administration or during follow-up. According to VAS, the mean value (SD) for PRGF® and BM-MSC with PRGF® went from 5 (1.8) to 4.5 (2.2) (p = 0.389) and from 5.3 (1.9) to 3.5 (2.5) (p = 0.01), respectively at 12 months. In WOMAC, the mean (SD) baseline and 12-month overall WOMAC scores in patients treated with PRGF® was 31.9 (16.2) and 22.3 (15.8) respectively (p = 0.002) while that for patients treated with BM-MSC plus PRGF® was 33.4 (18.7) and 23.0 (16.6) (p = 0.053). Although statistical significances between groups have been not detected, only patients being treated with BM-MSC plus PRGF® could be considered as a OA treatment responders following OARSI criteria. X-ray and MRI (WORMS protocol) revealed no changes in knee joint space width or joint damage.

**Conclusions:**

Treatment with BM-MSC associated with PRGF® was shown to be a viable therapeutic option for osteoarthritis of the knee, with clinical improvement at the end of follow-up. Further phase III clinical trials would be necessary to confirm the efficacy.

*Trial registration* Clinical Trials.gov identifier NCT02365142. Nº EudraCT: 2011-006036-23

## Background

Symptomatic knee osteoarthritis (OA) occurs in 10% of men and 13% of women aged 60 years or older and is increasing significantly because the aging population [1, 2]. To date, total knee arthroplasty is the definitive treatment for advanced grade of symptomatic OA [3].

Mesenchymal stromal cells (MSCs) offer some interesting properties that could be useful for treating OA [4]. Because OA is the result of an inflammatory pathophysiology, with the presence of inflammatory cells, the modulation of the immunological responses and the expression of inflammatory mediators by MSCs present a major therapeutic potential [4–7] providing the theoretical basis for the exogenous administration of MSC to be effective. It has been suggested that the number of MSC in subchondral bone decreases with age and correlates with OA degree, and that this deficit is necessary for the development of the degenerative process [8–13]. In addition, it has been proposed that during tissue injury the reparative process requires the migration of MSCs to the site of injury to exert their effects [14, 15].

Although treatment with MSCs in knee OA has been shown as a safe and feasible procedure in numerous phase I and II clinical trials, there are still doubts about its efficacy, mechanism of action, dosing and use of adjuvants [16–19]. Thus, the good preliminary results in safety have encouraged clinicians to conduct clinical trials focused on the efficacy of MSCs.

The use of platelet rich plasma (PRP) in orthopedic surgery is growing for the treatment of osteoarthritis [20, 21]. However, due to the different methods of production standardization is difficult and there is an open debate concerning the interpretation of the research on PRP [21, 22]. In any case, the identification of PRP-derived anabolic growth factors (basic FGF, TGF-β1, TGF-β2, EGF, IGF-I, PDGF-AB, PDGF-BB, VEGF) and anti-inflammatory cytokines (IL-1ra, sTNF-R1, sTNF-RII, IL-4, IL-10, IL-13, IFNγ) may present therapeutic potential in the treatment of osteoarthritis, especially in combination with MSCs, through enhancing or preserving their regenerative properties [23–25]. In addition, a positive effect on joint lubrication through stimulation of synoviocytes-derived hyaluronic acid has been reported for PRP, thus, when administered as co-adjuvant of MSCs, PRP could increase the intra-articular retention and survival of MSCs in an anti-inflammatory environment [26, 27].

Despite the existence and availability of these two promising biological treatments, little is known about the clinical effect of the combination of the two. Our aim was to assess the efficacy of intraarticular injection of 100 million bone marrow-derived MSCs (BM-MSCs) in combination with autologous PRP (PRGF®) in the treatment of OA.

## Materials and methods

This is a phase II randomized clinical trial with active control conducted between August 2016 and July 2018, involving the Clínica Universidad de Navarra (Pamplona, Spain); IBSAL-Hospital Universitario de Salamanca (Salamanca, Spain) and Hospital Vithas San José (Vitoria, Spain). All the procedures were approved by the Institutional Review Board of Navarra and the Spanish Agency of Medicines and Medical Devices (Nº EudraCT: 2011-006036-23, Clinical Trials.gov identifier: NCT02365142) an Ethics Commite for Clinical Research of Navarra, Salamanca and País Vasco. All participants provided written informed consent.

### Criteria for eligibility of patients

Inclusion criteria were males and females aged 18–80, diagnosis of knee OA according to American College of Rheumatology criteria, visual analogue scale (VAS) joint pain ≥ 2.5, Kellgren-Lawrence radiological classification scale ≥ 2, body mass index between 20 and 35 kg/m^2^, previous failed treatment with hyaluronic acid (HA) and availability for follow-up during the study period.

Exclusion criteria were: previous diagnosis of polyarticular disease, severe mechanical deformation (> 15° varus/valgus), systemic autoimmune rheumatic disease, arthroscopy or intraarticular infiltration in the last 6 months, chronic treatment with immunosuppressive or anticoagulant drugs, corticosteroids treatment in the 3 last months, non-steroidal anti-inflammatory therapy in the last 15 days, bilateral knee OA requiring treatment in both knees, poorly controlled diabetes mellitus, blood dyscrasias.

### Treatment groups

Patients were randomly assigned to receive either PRGF® or BM-MSC and PRGF® treatment with allocation as per a computer-generated randomization schedule. This allocation procedure was centralized to ensure that no center knew the treatment allocation of any patient until the patient had been recruited into the trial. The biopsy linked to the use of autologous cells did not allow any blinding procedure for patients nor clinicians for ethical reasons. Blinding processes were carried out for imaging evaluation and statistical analysis. The control group was constituted by patients who received PRGF® as a weekly single intra-articular injection of PRGF®, final volume of 8 ml, for 3 weeks. The BM-MSCs group was formed by patients who received a single intra-articular injection of 100 × 10^6^ autologous cultured BM-MSCs in 3 ml Ringer’s lactate solution, followed by an intraarticular injection of 8 ml of PRGF®. A weekly single intra-articular injection of PRGF®, in a final volume of 8 ml, was applied for 2 additional weeks.

### Sample size calculation

A sample size of 60 patients (30 patients per group) was required to achieve a 95% power to detect a Cohen’s effect size of 1, assuming a standard deviation for both groups of 10, an alpha value of 0.05 (two-sided test), and an expected dropout rate of 10%.

### Cell culture

BM-MSCs were generated under good manufacturing practice conditions (GMP) with standard operating procedures as previously described [28]. Briefly, bone marrow (100 ml) was harvested from the pelvic bone (iliac crest) under sterile conditions. The mononuclear cell fraction was isolated by Ficoll density gradient centrifugation (Ficoll-Paque, GE Healthcare Bio-Sciences AB, Uppsala, Sweden). Mononuclear cells, ranging between 20 × 10^6^ and 60 × 10^6^, were subsequently seeded in 175 cm^2^ flasks with growth medium, which consisted of αMEM without ribonucleosides (Gibco, Life Technologies, Carlsbad, CA, USA) supplemented with 5% platelet lysate, 2 units/ml heparin, penicillin–streptomycin at 1% (Gibco, Life Technologies) and 1 ng/ml human fibroblast growth factor (bFGF) (Sigma-Aldrich, St. Louis, MO, USA). The flasks were maintained in culture at 37ºC in 5% CO_2_ atmosphere. The growth medium was changed every 3–4 days. About 10–15 days later, colonies were formed, and the cells were split with TrypLE Select™ (Life Technologies) and seeded at 3000–5000 cells/cm^2^. Once 70–80% confluence was reached, cells were split again and cultured until they were available in the amounts required to be administered to patients. Finally, cells were harvested with TrypLE Select™, washed three times with PBS and resuspended in Ringer’s lactate buffer (Grifols, Barcelona, Spain) containing 1% human albumin (Grifols), to be administered within 24 h of harvesting of the cells. Cells were characterized according to ISCT criteria. Cells were then analyzed by flow cytometry (FACSCalibur, BD Biosciences, San José, CA, USA) with the appropriate antibodies (BD Biosciences) to confirm expression of surface markers CD90, CD73 and CD44, as well as absence of CD34 and CD45.

### PRGF® preparation

PRGF® was obtained with PRGF-Endoret (BTI System II; BTI Biotechnology Institute, Vitoria, Spain) by centrifugation at 580×*g* for 8 min at room temperature. The top volume of plasma, with a platelet count similar to peripheral blood, was not used. The plasma fraction, located just above the sedimented red blood cells but not including the buffy coat, was collected in another tube. This plasma contains a moderate enrichment in platelets (two to threefold the platelet count of the peripheral blood) with no leukocytes. Thereafter, in the injection room 10% Calcium Chloride was added to activate platelets (0.05 ml of calcium chloride per ml PRP).

### Cell and PRP injection

Cell and PRGF® injection were performed through a lateral patellar approach as previously reported [18]. For cell treatment, cell injection was applied 3–4 weeks after the iliac crest biopsy had been performed. In all of the patients, cells were administered within the first hour after harvesting. For this purpose, a 19 G needle was used in two consecutive intraarticular injections. In the first one, 100 × 10^6^ BM-MSCs were administered in 3 ml of Ringer lactate. Subsequently, 8 ml of PRGF® were injected using the same route. In the PRGF®-only group, 3 separate doses were applied, one week each, 8 ml of PRP per injection.

### Outcomes of interest

The clinical response to intra-articular infusion of both treatments was assessed using the following procedures:

To clinically assess pain and function, two scale-based methods, Visual Analog Scale (VAS) and the Likert version of the Western Ontario and McMaster Universities Osteoarthritis Index (WOMAC), were evaluated at baseline, 3, 6 and 12 months after treatment [29, 30]. VAS ranges from 0 (maximum relief, i.e. no pain) to 10 (no relief, i.e. maximal pain). WOMAC comprises three sub scores, pain, which includes 5 items; stiffness, with 2 items; and physical function, with 17 items. According to Osteoarthritis Research Society International (OARSI), patients were considered WOMAC responders when they reported an improvement of 20% on at least 2 items together with an improvement of 10 points in the overall scale [31].

To provide a radiographic assessment of the joint space width, Rosenberg X-ray projections were taken at baseline and 12 months afterwards. A custom methacrylate patient positioner was used to achieve a comparative view as previously described [28].

A Magnetic Resonance Imaging (MRI) study was carried out at baseline and 12 months after treatment. One experienced radiologist evaluated MRI images in a blinded manner by assessing the number and location of the lesions, cartilage thickness, signal intensity, and subchondral bone alteration and volume, following the Whole-Organ Magnetic Resonance Imaging Score (WORMS) protocol, in which higher score values indicate more damage [32]. Three Tesla Magnetom TRIO equipment (Siemens, Erlangen, Germany) was used following a protocol which included an axial T1 weighted spin‐echo (SE) (TR 700 ms/TE 11 ms) slice thickness (ST) of 4 mm; coronal T1 weighted SE (TR 700 ms/TE 11 ms) ST of 4 mm; SPACE sagittalT1 weighted (TR 800 ms/TE 43 ms) ST of 0.7 mm; 2D Intermediate weighted (IW) Fat Saturation (FS) Sagittal (TR 4620 ms/TE 21 ms) ST 3 mm; Coronal 2D IW (TR 4370 ms/TE 11 ms) ST 3 mm and sagittal T2* 2D (MapIt TR 958 ms, TE 4, 11, 18, 25 y 32 ms) ST 3 mm.

### Statistics

The predefined analysis strategy was by intention to treat. Data were summarized using means and standard deviations (SD), medians and percentiles 25 (p25) and 75 (p75), and counts and percentages. The Shapiro–Wilk test was used to test the normality assumption. For comparisons, we used the unpaired Student’s t test, the Mann–Whitney U test, the paired Student’s t test, and the Wilcoxon matched-pairs signed-ranks test, as appropriate. All tests were two-tailed. A p value < 0.05 was considered statistically significant. All the statistical analyses were performed using Stata 14 (Stata Corp. 2015. Stata Statistical Software: Release 14. College Station, TX: Stata Corp LP).

## Results

### Demographic data

**Fig. 1:**
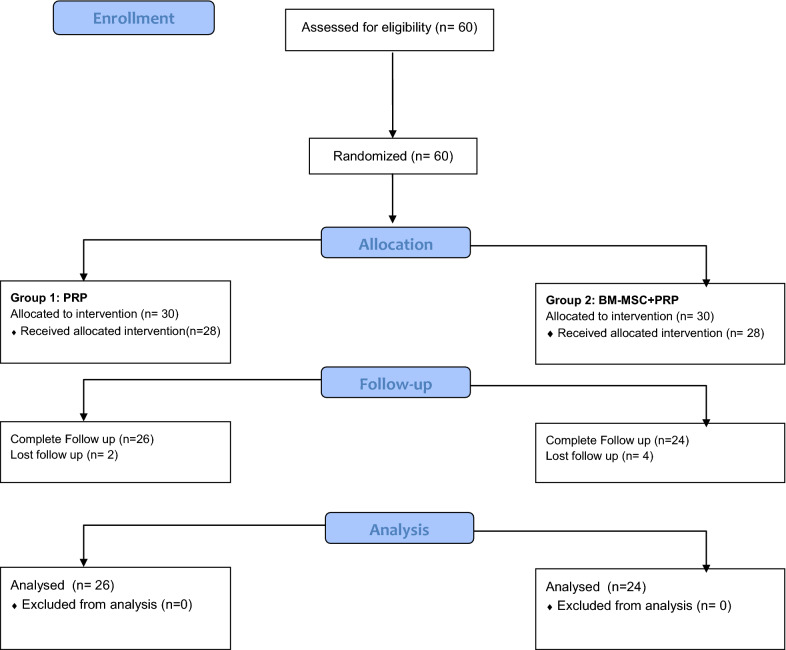
Study flow diagram. Patients were screened in the different participating centers using the inclusion and exclusion criteria

Sixty patients were assessed for eligibility and were consecutively randomized to treatment groups (Fig. 1). Four patients who had been randomly assigned to the PRGF® group and 6 patients in the group of BM-MSC plus PRGF were excluded from the analysis because of withdrawal of consent (n = 3), loss of follow up (n = 6) and detection of trisomy in the culture (n = 1). In total, 26 patients completed follow up in the PRGF® group and 24 in the BM-MSC plus PRGF® group, and all were analyzed. Both groups showed similar baseline characteristics of age, body mass index and OA severity according to the Kellgren–Lawrence scale (Table 1).

**Table 1:** Baseline characteristics of patients

	PRGF	BM-MSCs + PRGF
N	26	24
Age (range)	54.6 (33,70)	56 (40, 62)
Males, n (%)	16 (61.54)	17 (29.17)
BMI (kg/m^2^)	25.3 (23.1, 28.7)	27 (25.2, 29.3)
K-L 2, n (%)	8 (30.77)	5 (20.83)
K-L 3, n (%)	5 (19.23)	2 (20)
K-L 4, n (%)	13 (50)	12 (50)

Unless specified, data are presented as mean. OA, osteoarthritis; K–L, Kellgren and Lawrence grading scale of severity of knee OA

### Adverse events

No serious adverse events or complications derived from the procedures or treatments were noted. Articular pain requiring anti-inflammatory treatment during the first 24 h after infiltration was observed in six patients in BM-MSC plus PRGF® group (five after the administration of BM-MSC plus PRGF and one after the third PRGF® injection). All patients recovered completely without sequelae.

### Clinical effects

The assessment of pain by VAS showed an improvement in pain when compared with baseline in both groups. This improvement in pain level was more evident for the BM-MSC plus PRGF® group. The mean (SD) baseline and 12-month VAS scores in patients receiving PRGF® was 5 (1.8) and 4.5 (2.2), respectively (p > 0.05). For the BM-MSC plus PRGF®, the mean (SD) baseline and 12-month VAS scores were 5.3 (1.9) and 3.5 (2.5), respectively (p = 0.01). The median (p25; p75) of percentage reduction in VAS from baseline to 12 months was 39.2% (− 67%; 0%) for BM-MSC plus PRGF® group whereas it was 12.5% (− 29%; 40%) for PRGF® group (p = 0.082) (Table 2). Although this improvement was higher with the BM-MSC plus PRGF® treatment, the differences in pain assessment by VAS did not show statistically significant differences between PRGF® and BM-MSC plus PRGF® at any time points. Similar results were observed in the WOMAC score with an improvement in the overall WOMAC score observed at the different time points in comparison with the baseline value in both groups (Table 3). The mean (SD) baseline and 12-month overall WOMAC scores in patients treated with PRGF® were 31.9 (16.2) and 22.3 (15.8), respectively (p = 0.002) and 33.4 (18.7) and 23.0 (16.6) for patients treated with BM-MSC plus PRGF® at baseline (n = 24) and 12-month (n = 21) respectively (p = 0.053). The median (p25; p75) WOMAC score reductions from baseline to 12 months were 37.3% (− 80%; 8%) and 23.4% (− 63%; 6.5%) for BM-MSC plus PRGF® group and PRGF® group, respectively with no significant differences between groups (p = 0.691). The Osteoarthritis Research Society International (OARSI) defined treatment response in patients with OA is defined as an improvement greater than 20 percent on at least 2 items evaluated at 12 months and a difference greater than 10 points overall. According to this definition and the results showed, only patients being treated with BM-MSC plus PRGF® could be considered as treatment responders.

**Table 2:** VAS before administration of treatments and 3, 6 and 12 months afterwards

Time	PRP	BM-MSCs + PRP
Baseline	5 (1.8)	5.3 (1.9)
3 months	3.8 (1.6)*	3.8 (2)*
6 months	3.5 (2)*	3.3 (2.2)*
12 months	4.5 (2.2)	3.5 (2.5)**

Data are presented as mean (SD). Intragroup comparisons between follow-up values and baseline values: *p < 0.05; **p = 0.01

**Table 3:** WOMAC scores before administration of treatments and 3, 6 and 12 months afterwards

WOMAC	Time	PRGF	BM-MSCs + PRGF
Pain	Baseline	6.6 (2.9)	6.6 (4.4)
	3 months	4.3 (3.3)**	4.6 (3.3)*
	6 months	4.4 (3)**	4.3 ( 3.6)
	12 months	4.5 (3.2)**	4.1 (3.6)
	Improvement (%)	27.3	45.5
Stiffness	Baseline	3 (1.6)	3.3 (2.1)
	3 months	1.9 (1.4)**	2.3 (2.2)**
	6 months	2.2 (1.6)	2 (1.9)*
	12 months	2.1 (1.6)*	2.1 (1.9)
	Improvement (%)	33.3	50
Physical function	Baseline	22.3 (12.8)	23.5 (13.2)
	3 months	15.5 (12.6)**	17.6 (12.6)*
	6 months	16.3 (11.1)*	14.9 (11.8)*
	12 months	15.5 (11.9)**	16.7 (11.6)*
	Improvement (%)	24.4	28.6
Overall	Baseline	31.9 (16.2)	33.4 (18.7)
	3 months	21.7 (17.1)**	24.4 (17.4)*
	6 months	23 (15)**	21.3 (16.6)*
	12 months	22.3 (15.8)**	23.0 (16.6)^**#**^
	Improvement (%)	23.4	37.3

At baseline, 3 months, 6 months and 12 months, data are presented as mean (SD). Intragroup comparisons between follow-up values and baseline values of WOMAC score: *p < 0.01; **p < 0.05, ^**#**^p = 0.05. Improvement (%) is the median improvement in percentage between the basal value and 12 months

### Radiological and MRI findings

The X-rays analysis of the knee joint space during follow up did not show any clinical effect (Additional file 1: Table S1). Because fifty per cent of the patients have a knee joint space baseline value of 0 mm (no joint space), detection of a clinical effect by X-ray imaging has certain limitations. Consistent with the X-ray results, the analysis of the MRI following the WORMS protocol did not show any significant change during follow up (Additional file 2: Table S2).

## Discussion

We present the results of a phase II clinical trial that followed a previous phase I clinical trial focused primarily on safety and feasibility, with promising secondary efficacy results using a dose of autologous 100 million BM-MSCs, and with a clinical improvement that remained in long-term follow-up [18, 33].

The use of co-adjuvants with BM-MSCs in the treatment of OA is a topic of interest. Some authors report functional and clinical improvement without the use of coadjuvants, and others report clinical improvement with the use of HA as an adjuvant for BM-MSCs, while the use of PRP as adjuvant is still controversial [28, 33, 34]. The clinical results published without co-adjuvants seems to be comparable with our results using BM-MSC and PRGF®. However, further analysis are needed in that sense.

Preclinical studies showed that PRP could be used as a potent and reliable chondrogenic inducer as a substitute for TGF-β1. Some of its properties, such as its low immunogenicity, easy accessibility and role as an activator of the mechanism of hyaline cartilage production (related with the TGF-β1/SMAD signaling pathway) define the PRP as a potentially attractive coadjuvant [35, 36]. Reporting a clinical study with 18 patients, Bastos et al. concluded that the use of PRP as co-adjuvant together with 40 million BM-MSCs did not provide additional benefits over the use of BM-MSCs alone [37]. The same group, in a recent clinical trial, compared the use of 40 million BM-MSCs (n = 14) with and without PRP (n = 16) and corticosteroids (n = 17) as standard control treatment, no differences were observed in the use of adjuvant therapy with PRP in the treatment of these patients [17]. Interestingly, Bastos et al. reported the use of a white blood cell-depleted PRP (< 0.3% from the initial red blood cells and leukocytes were present) containing between 5.4- to 7.3-fold increase in platelet concentration (1.4 × 10^6^ to 1.9 × 10^6^ platelets/μl). It differs in some aspect to PRGF® which presents a moderate enrichment in platelets (two to threefold the platelet count of the peripheral blood) with no leukocytes. In consequence, the use of different products and the adjuvant effect over the BM-MSC could not be the same and the difference observed in our study could be a consequence of these variations [37].

PRGF® is a type of PRP that presents a number of characteristics that differentiate it from other blood products. The main characteristics that define it are its concentration of platelets two to threefold higher than the levels in blood and the absence of leukocytes. This makes PRGF® a type of PRP that is poor in leukocytes, which are increasingly recommended for treating pathologies such as osteoarthritis, since promotes extracellular matrix repair, decreases inflammation, and slow-down joint degeneration [38].

Furthermore, the presence of leukocytes leads to an increase in the levels of proinflammatory molecules that can influence not only the development of the pathology [39] but also harm the MSCs that are administered [40]. In an in vivo study, Dregalla et al. compared the response of bone marrow derived human mesenchymal stem cells cultured in different types of PRP. The authors observed that PRP without leukocites increased cell proliferation and hyaluronic acid synthesis compared with leukocyte-rich PRP [41].

The use of PRGF® with adjuvant cell products was studied in dogs with hip osteoarthritis which received PRGF® and adipose-derived mesenchymal stem cell. The results demonstrated that the treatment was safe and effective, significantly improving aspects such as peak vertical force and vertical impulse [42].

As in our previous clinical trial, we detected no regenerative effect after the administration of BM-MSCs, even after the use of T2-Mapping sequence. We were capable of detecting positive clinical effects when pain was evaluated. In that sense, Caplan et al*.* proposed a change in the name of MSCs from mesenchymal stromal cells to medicinal signaling cells [4, 43]. This change in nomenclature is explained because paracrine effects of MSCs on inflammatory cells, and recently a decreased level of inflammatory cytokines (IL-17α, IL-10 and TNF-α) with the administration of BM-MSC have been reported [17]. Futures studies should focus on the lower OA grades to delimit the paracrine effects of biological treatments in chondrocytes. We should note that 50% of the patients presented the higher degree of OA (grade IV), so the expected combined paracrine effect of PRGF® and BM-MSCs over the chondrocytes was not predictable, due to the scarce number of viable chondrocytes in cartilage with advanced OA.

Our study had several limitations. The interventions could not be blinded for patients nor clinicians for ethical reasons. Efforts to minimize potential biases were made using blinded imaging evaluators and a blinded process for statistical analysis. Although placebo effect may explain some degree of the treatment impact, it does not seem plausible that this phenomenon could entirely explain the clinical improvement of patients receiving BM-MSC plus PRGF®. Some authors have defined the placebo effect as a reduction of 1.37 points on the VAS scale and 9 on the WOMAC [44, 45]. We report here, at 12 months, that the decrease in the VAS scoring was 1.8 points, and 10.4 in WOMAC score. Saltzman et al*.* in a meta-analysis published in 2016 objectified the effect of the saline injection on the VAS and WOMAC scales at six months. They included 14 cohorts of 13 different studies in knee OA where patients were treated with a saline injection. A saline serum effect of 1.66 in VAS and 11.34 in WOMAC at six months was determined [46]. In our study, at 6 months, we detected a decrease on the VAS of 2.0 points and 12.1 points on the WOMAC in BM-MSC plus PRGF®. In addition, patients in the group receiving BM-MSCs plus PRGF® were the only ones that can be considered responders to the treatment, according to the definition of responder to treatment for osteoarthritis of the OARSI with an improvement greater than 20 percent on each item (the OARSI definition highlights an improvement of 20% on at least 2 items) evaluated at 12 months and a difference greater than 10 points overall. Overall, these results are consistent in the literature with the use of both allogeneic and autologous cultured BM-MSCs [6, 16, 19, 28, 34, 47].

## Conclusion

Treatment with BM-MSC associated with PRGF® has been shown to be a viable therapeutic option in the treatment of OA of the knee, resulting in improvement at the end of the follow-up in VAS and WOMAC score. Only patients being treated with BM-MSC plus PRGF® could be considered as OA treatment responders. PRGF® alone showed improvement in WOMAC but not in VAS score, however, magnitude clinical differences assessed between groups were not statistically significant. Therefore, further phase III clinical trials would be necessary to confirm the efficacy of the treatment.

## Supplementary information

**Additional file 1: Table S1.** X-ray measurement of the evolution of the knee articular interline and 12 months after the administration of treatments.

**Additional file 2: Table S2.** WORMS score before administration of treatments and 12 months afterwards.

## Data Availability

All the data presented is available upon request.

## Abbreviations

αMEM: Alpha minimum essential medium
bFGF: Fibroblast growth factor
BM-MSCs: Bone marrow mesenchymal stromal cells
GMP: Good manufacture practices
ISCT: International Society for Cellular Therapy
K–L: Kellgren and Lawrence scale
MRI: Magnetic resonance imaging
MSCs: Mesenchymal stromal cells
OA: Osteoarthritis
VAS: Visual analogue scale
WOMAC: Western Ontario and McMaster Universities Osteoarthritis Index
WORMS: Whole-Organ Magnetic Resonance Imaging Score
