# Supplementary material for: Phase II multicenter randomized controlled clinical trial on the efficacy of intra-articular injection of autologous bone marrow mesenchymal stem cells with platelet rich plasma for the treatment of knee osteoarthritis
Source: J Transl Med. 2020 Sep 18;18:356. doi: 10.1186/s12967-020-02530-6 (PMC7501623; doi:10.1186/s12967-020-02530-6)
Supplement: Supplementary file 1 — Additional file 1: Table S1. X-ray measurement of the evolution of the knee articular interline and 12 months after the administration of treatments. [file 12967_2020_2530_MOESM1_ESM.docx]

**Table S1.** X-ray measurement of the evolution of the knee articular interline and 12 months after the administration of treatments

| **Time** | **PRGF** | **BM-MSCs + PRGF** |
| --- | --- | --- |
| **Baseline** | 2.06 (2.17) | 2.04 (1.93) |
| **12 months** | 1.77 (1.97) | 1.41 (1.96) |

For each group of treatment, variation for knee joint space width, which was measured in mm, was calculated by subtracting, for each patient of the group, the value at 6 or 12 months from the baseline value. Data are presented as the median (IQR) of each group.
