# Supplementary material for: Phase II multicenter randomized controlled clinical trial on the efficacy of intra-articular injection of autologous bone marrow mesenchymal stem cells with platelet rich plasma for the treatment of knee osteoarthritis
Source: J Transl Med. 2020 Sep 18;18:356. doi: 10.1186/s12967-020-02530-6 (PMC7501623; doi:10.1186/s12967-020-02530-6)
Supplement: Supplementary file 2 — Additional file 2: Table S2. WORMS score before administration of treatments and 12 months afterwards. [file 12967_2020_2530_MOESM2_ESM.docx]

**Table S2.** WORMS score before administration of treatments and 12 months afterwards

| **Time** | **PRGF** | **BM-MSCs+PRGF** |
| --- | --- | --- |
| **Baseline** | 73.8 (30.9) | 74.4 (28) |
| **12 months** | 77.4 (31.5) | 79.8 (29.1) |

The overall WORMS scores at baseline and 12 months afterwards are presented as the media (SD) of each group.
